# *HLF gene* is a poor prognostic factor in acute myeloid leukemia patients with *FLT3-*ITD/NPM1 mutations undergoing hematopoietic transplantation

**DOI:** 10.1371/journal.pone.0333690

**Published:** 2025-10-14

**Authors:** Xiaoyu Xu, Xinxin Ge, Airui Jiang, Jia Chen, Qiaocheng Qiu, Zixuan Ding, Mingqing Zhu, Jinlan Pan, Zixing Chen, Wenwen Du, Depei Wu, Suning Chen, Hongjie Shen

**Affiliations:** 1 Jiangsu Institute of Hematology, National Center of Hematological Clinical Medicine Research, NHC Key Laboratory of Thrombosis and Hemostasis of Ministry of Health, the First Affiliated Hospital of Soochow University, Suzhou, China; 2 Collaborative Innovation Center of Hematology, Institute of Blood and Marrow Transplantation, Suzhou, China; 3 Department of Pulmonary and Critical Care Medicine, the First Affiliated Hospital of Soochow University, Suzhou, China; European Institute of Oncology, ITALY

## Abstract

Acute myeloid leukemia (AML) patients with *FLT3*-ITD mutations were benefit from hematopoietic cell transplantation (HSCT) in the first complete remission. Previous research suggested that newly diagnosed AML patients with high allelic ratio (AR) of *FLT3*-ITD have unfavorable survivals, while newly diagnosed AML patients with lower *FLT3*-ITD AR and concomitant *NPM1* mutations have favorable outcomes. In AML patients with *FLT3*-ITD, co-occurrence with *DNMT3A*, and *NPM1* mutations (triple-mutated AML patients) have the worst prognoses, however, it is little known about how these mutations synergize in these triple-mutated AML patients. Here we showed that hepatic leukemia factor (*HLF*) gene was more highly expressed in triple-mutated AML patients than in those without the *DNMT3A* mutations. We found that *HLF* gene expressions had significant difference in triple-mutated and *FLT3-*ITD/*NPM1* AML patients (double-mutated AML patients). Moreover, in *DNMT3A* mutated AML patients, correlated with high *HLF* gene expression, which may be itself associated with poor survival rate and drug resistance. Overall our data establish that *HLF* gene as a novel biomarker in this genetically defined the triple-mutated AML subgroup.

## Introduction

Acute myeloid leukemia (AML), which results from the blockage of differentiation and chaotic proliferation of immature myeloid progenitors, is a genetically diverse and aggressive hematological malignancy [1]. Despite advances in leukemia therapy and considerable rates of remission, 40–50% of AML patients experience short-term recurrence accompanied by a heaeareduced treatment response [2]. Activating internal tandem duplication of FMS-like tyrosine kinase 3 (*FLT3*-ITD) is one of the most frequently mutations in AML patients with a frequency of 20–30% and associated with an increased risk of adverse outcome [3,4]. The *FLT3-*ITD mutations are associated with a poorer prognosis and a higher rate of relapse after a short duration of remission compared with other AML subtypes [5,6]. Although *FLT3* inhibitors demonstrate single-agent efficacy by inducing complete remission (CR), the utility of these inhibitors has been hampered by acquired resistance with secondary TKD mutations, illustrating the need for additional therapeutic targets [7].

*NPM1* encodes a shuttling phosphoprotein between the nucleus and cytoplasm, plays a vital role in cell proliferation and apoptosis [8]. Isolated *NPM1* mutations leading to cytoplasmic localization of npm1 protein, confer a high complete response rate, and improve event-free survival rate (EFS) and overall survival rate (OS) in patients with normal karyotype AML resulting in outcomes similar to patients with favorable cytogenetics [9,10]. The *NPM1* mutations frequently coexist with *FLT3-*ITD, *DNMT3A* mutations.

*DNMT3A*, which catalyzes the addition of methyl groups to the cytosine residue of CpG dinucleotides in DNA, frequently occurs in normal karyotype AML and associated with monocytic AML [11]. Which regulates gene expression by recruiting proteins involved in gene repression or by inhibiting the binding of transcription factor to DNA. There are conflicting data regarding the prognostic effect of *DNMT3A* on outcome of AML with either no or negative effect [12]. Mutations in *DNMT3A* gene alter the gene’s normal function and play an important role in AML prognosis [13]. *DNMT3A* mutations induced hematopoietic stem cell expansion, cooperated with *FLT3-*ITD and *NPM1* mutations to induce AML in vivo, and promoted resistance to anthracycline chemotherapy. In AML patients, the presence of *DNMT3A* R882 mutations predict minimal residual disease, underscoring their role in AML chemoresistance [14]. *DNMT3A* R882 mutations play a crucial role in driving AML chemoresistance, but the mechanisms of chemoresistance in AML are still unclear.

Hepatic leukemia factor (*HLF*) is a transcription factor belonging to the proline and acidic amino-acid-rich (PAR) basic leucine zipper (bZip) family [15]. Members of PAR bZIP family, including *HLF,* thyrotropin embryonic factor (TEF) and albumin D-site-binding protein (DBP), regulate the expression of genes involved in detoxification, drug metabolism and circadian rhythm control [16]. *HLF* gene is vital in the development and maintenance of hematopoietic stem cells (HSCs), contributing to the maintenance of HSC quiescence and self-renewal capacity. By preserving HSCs in a dormant state, *HLF* protects them from radiation- or chemotherapy-induced injury, thereby supporting long-term hematopoietic regeneration. In the context of hematopoietic malignancies, *HLF* overexpression has been linked to enhanced drug resistance through activation of survival pathways and detoxification-related gene programs in acute lymphoblastic leukemia [17]. These properties suggest that *HLF* may contribute to treatment refractoriness and disease persistence in aggressive leukemia subtypes.

Although its role in acute myeloid leukemia remains poorly understood, the molecular properties of *HLF* suggest that it could influence leukemic stem cell survival, therapeutic response, and disease persistence. Given that *HLF* is not routinely assessed in AML and remains largely unfamiliar to clinicians and researchers, a deeper understanding of its biological function and prognostic significance is warranted. The primary objective of this study was to evaluate the prognostic significance of *DNMT3A* mutation in patients with triple‐mutated AML. Additionally, we aimed to characterize the transcriptomic profile of these patients to identify genes of interest, with a particular focus on *HLF*, which may warrant further investigation as a potential therapeutic target.

## 2. Materials and methods

### 2.1. Clinical patients

A total of 84 AML patients with *FLT3-*ITD mutations were obtained in our study if they met the following criteria: (1) aged 18 years or older with a diagnosis of AML excluding acute promyelocytic leukemia according to the WHO 2016 classification of myeloid neoplasms and acute leukemia; (2) harboring triple mutations of *FLT3-ITD, NPM1*, and *DNMT3A*; (3) received two or three courses of standard chemotherapy followed by immediate allogeneic hematopoietic stem cell transplantation (HSCT); and (4) had complete clinical, laboratory, and follow-up data available. Patients were excluded if they died from acute graft-versus-host disease (aGVHD) before relapse assessment, had incomplete mutational or survival data, or received HSCT from multiple donors or underwent tandem transplantations [18], The patients were treated between September 22, 2011 to November 27, 2020 at First Affiliated Hospital of Soochow University. The study was approved by the Ethics Committee of the First Affiliated Hospital of Soochow University (No.747 of 2025 LSP (application)) and was conducted following the Declaration of Helsinki. The ethical approval included permission for genomic analyses. Major (78/84, 92.9%) patients were treated with standard “3 + 7” regimen for initial induction therapy (darubicin/ idarubicin + cytarabine). In elderly and severe underlying diseases patients, pre excitation scheme (cytarabine + aclarubicin + granulocyte-colony stimulating factor) were administered. The first consolidation therapy was generally the same as that used to achieve CR or high/medium-dose cytarabine at 2–3g/m^2^ were administered for consolidation therapy.

### 2.2. DNA sequencing and mutation analysis

Genomic DNA was extracted from bone marrow or peripheral blood samples at the onset of disease diagnosis by using Invitrogen DNA Extraction Kit. The mutational hotspots or whole coding regions of 51 genes (S1 Table) that recurrently mutated in hematological malignancies were sequenced. The procedures were in accordance with an amplicon-based Next Generation Sequencing (NGS) protocol with Ion Torrent PGM sequencer (Thermo Fisher Scientific, Waltham, MA, USA). An allele frequency threshold of 1% was defined for mutation detection. All *FLT3*-ITD mutations were confirmed by Sanger sequencing. Bone marrow or peripheral blood samples in CR or fingernail samples were also detected for exclusion of *FLT3*-ITD germline mutations.

### 2.3 *FLT3-ITD* improved method

All patients provided written informed consent according to the declaration of Helsinki. Bone marrow samples were examined a major panel of 49 genes mutation by NGS and *FLT3*-ITD AR by capillary electrophoresis in newly diagnosis. *FLT3*-ITD AR were evaluated in bone marrow samples directly after secondary chemotherapy. *FLT3-*ITD improved method, the same as previously method except for 40 cycles amplification of *FLT3*-ITD fragment, was used to evaluate *FLT3*-ITD VAF (variant allele frequency) after consolidation chemotherapy. The limit of *FLT3*-ITD VAF by improved method was set at 0.1% (S2 and S3 Tables).

### 2.4 RNA sequencing

RNA was extracted from 14 bone marrow samples of *FLT3-*ITD AML cases using Trizol reagent. These libraries were set up through TruSeq Stranded mRNA LT Sample Prep Kit (Illumina, San Diego, CA, USA). Then these libraries were detected on the Illumina sequencing platform (HiSeq TM 2500 or Illumina HiSeq X Ten) and 125 bp/150 bp paired-end reads were amplificated.

### 2.5 Statistical analysis

Overall survival (OS) is defined as the time from diagnosis to death or to the time of the last follow-up. Event free survival (EFS) is defined as the time from diagnosis to relapse, death or the time of last follow-up. Relapse-free survival (RFS) is defined as the time from transplantation to relapse, death or the time of last follow-up. All alive patients were followed on August 31, 2021. CR according to the European Leukemia Net (ELN) 2017 recommendations, was defined as: less than 5% blasts in the bone marrow, absence of circulating blasts and extramedullary disease, absolute neutrophil count (ANC) >1.0 × 10⁹/L, platelet count > 100 × 10⁹/L, and full recovery of peripheral blood counts without transfusion support.

The SPSS software (version 23.0; SPSS Inc., Chicago, IL) was applied in statistical analysis. The significance between categorical data was calculated by Chi-square test. For numerical data, independent samples t-tests were performed when homogeneity of variance was met, whereas the rank-sum test was used when variances were unequal. Kaplan-Meier method was employed for OS analysis, and log-rank test was used to compare differential survival rates among groups. A two-sided P < 0.05 was considered as statistical significance.

## 3. Results

### 3.1. Clinical characteristics

Our research summarized the clinical characteristics of 84 *FLT3*-ITD mutated AML patients. The median age of these patients was 37.5 years old (range, 18–60 years old), including 44 male and 40 female patients. The median WBC and PLT count were 31.3 × 10^9^/L (range, 1–431.91 × 10^9^/L) and 42.5 × 10^9^/L (range, 4–631 × 10^9^/L), while the median bone marrow blast cell percentage was 67.5% (range, 20–96%).

In those patients, *NPM1* mutations were the most frequently observed additional mutations, occurring in 41.7% (35/84) patients. Other common mutated genes were *DNMT3A* (22/84), *CEBPA* (12/84), and *RUNX1* (10/84). All mutations were evaluated in multiple databases (S4 Table). Only 24 (28.5%) patients demonstrated abnormal karyotype, among which t (8;21) were the most commonly seen aberrations. In selected 84 *FLT3-*ITD mutated AML patients, *FLT3*-ITD mutations were commonly detected with *NPM1*, *DNMT3A*, *CEBPA* and *RUNX1* mutations.

### 3.2 Chemotherapy treatment results

In *FLT3-*ITD mutated AML patients, *DNMT3A* mutated group had lower CR rate after one standard chemotherapy than that of group without *DNMT3A* mutations (X^2 ^= 3.207, P = 0.073). In *FLT3-*ITD mutated AML patients without *DNMT3A* mutations 58 (93.5%) got CR after two courses of chemotherapy, while only 17 in 22 patients got CR after two courses chemotherapy in these patients with *DNMT3A* mutations; there was statistical difference between these two groups (fisher, P = 0.049). In *FLT3*-ITD mutated AML patients, *DNMT3A* mutated group had higher ITD positive rate after two standard chemotherapy than that of group without *DNMT3A* mutations (fisher, P < 0.001, Table 1). The CR rate of t (8; 21) patients, was like *CEBPA* double mutation patients, two patients didn’t get CR after one course therapy (CR1) and all got CR after two courses therapy (CR2). All *CEBPA* double mutated patients got CR1. At mean time, ITD positive rate were both 0 after two courses therapy (Table 1).

**Table 1:** CR and ITD positive number of 84 *FLT3*-ITD mutated AML patients.

Group	Number	CR number*	ITD positive number
*RUNX1-RUNX1T1*	7	7	0
*CEBPA* double mutation	10	10	0
*NPM1* without *DNMT3A* mutation	17	17	2
*DNMT3A* mutation	22	17	11

* complete remission number after two courses therapy.

### 3.3 Transplantation outcomes and multivariate analysis

Univariate Cox proportional hazard regression analysis for OS showed that there were significant difference in OS of relapse, *DNMT3A* mutations, PLT cell counts, one course CR (CR1) and two courses CR (CR2), which were further included in multivariate Cox regression analysis (Table 2). Univariate Cox proportional hazard regression analysis for EFS showed that there was significant difference in *DNMT3A* mutations, *RUNX1* mutations, CR1 and CR2, which were further included in multivariate Cox regression analysis (Table 3). In the stepwise forward Cox model for OS, relapse was the strongest independent predictor in the first step (HR = 0.018, 95% CI: 0.004–0.078, P < 0.001). In the final model, both relapse (HR = 0.008, 95% CI: 0.001–0.047, P < 0.001) and PLT count (HR = 1.012, 95% CI: 1.006–1.017, P < 0.001) remained significant, indicating that absence of relapse was strongly protective, whereas higher platelet counts were associated with increased mortality risk. For RFS, achievement of CR2 (HR = 0.115, 95% CI: 0.045–0.296, P < 0.001) remained a significant protective factor in the first step; in the final model, both CR1 (HR = 0.168, 95% CI: 0.053–0.529, P = 0.002) and CR2 (HR = 0.260, 95% CI: 0.097–0.696, P = 0.007) were retained, highlighting the strong protective effect of achieving complete remission.

**Table 2:** Univariate and multivariate Cox proportional hazard regression analysis for OS.

OS	HR	Exp	P	HR	Exp	P
Age	1.024	0.988-1.062	0.192			
Sex	1.745	0.740-4.117	0.204			
relapse	56.062	12.801-245.528	<0.001*	0.008	0.001–0.047	<0.001*
*NPM1*	1.419	0.626-3.217	0.402			
*DNMT3A*	3.601	1.585-8.180	0.002*			
*CEBPA*	0.037	0.000-3.811	0.163			
*RUNX1*	2.642	0.959-7.277	0.06			
Blast	1.011	0.990-1.032	0.308			
WBC	0.997	0.991-1.003	0.322			
Hb	1.006	0.987-1.026	0.545			
PLT	1.006	1.002-1.009	0.001*	1.012	1.006–1.017	<0.001*
Fusion gene	0.036	0.000-3.459	0.154			
CR1	0.195	0.077-0.498	0.001*			
CR2	0.157	0.063-0.393	<0.001*			

OS: overall survival; NPM1: presence of NPM1 mutation at initial diagnosis; CR1: the patient achieved complete remission (CR) after initial induction therapy, based on the 2017 ELN criteria; CR2: complete remission after two courses therapy, based on the 2017 ELN criteria; *P < 0.01.

**Table 3:** Univariate and multivariate Cox proportional hazard regression analysis for EFS.

EFS	HR	Exp	P	HR	Exp	P
Age	1.022	0.986-1.060	0.234			
Sex	1.675	0.703-3.994	0.245			
*NPM1*	1.349	0.583-3.124	0.484			
*DNMT3A*	3.585	1.551-8.286	0.003*			
*CEBPA*	0.037	0.000-4.293	0.174			
*RUNX1*	3.570	1.384-9.209	0.008*			
Blast	1.013	0.992-1.034	0.236			
WBC	0.999	0.994-1.004	0.659			
Hb	1.004	0.984-1.024	0.706			
PLT	1.001	0.996-1.005	0.773			
Fusion gene	0.036	0.000-3.866	0.164			
CR1	0.123	0.041-0.364	<0.001*	0.168	0.053–0.529	0.002*
CR2	0.115	0.045-0.296	<0.001*	0.260	0.097–0.696	0.007*

EFS: event free survival; NPM1:presence of NPM1 mutation at initial diagnosis; CR1: the patient achieved complete remission (CR) after initial induction therapy, based on the 2017 ELN criteria; CR2: complete remission after two courses therapy, based on the 2017 ELN criteria; *P < 0.01

**Fig 1:**
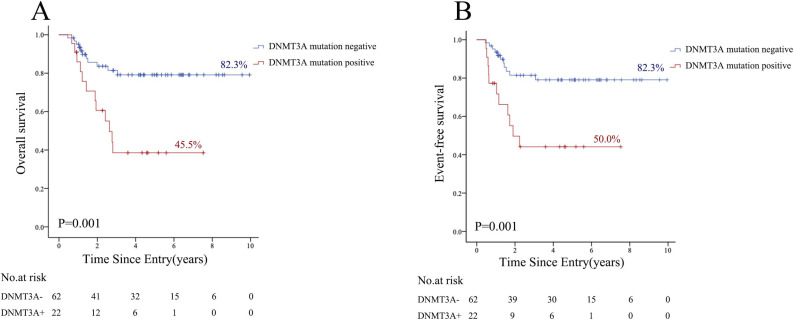
OS. (A) and EFS (B) by competing risk analysis for DNMT3A mutations.

*RUNX1* mutations, seen in 14.2% *FLT3-*ITD mutated AML cases, did not impact the clinical outcome of these patients (P = 0.06), but effected EFS (P = 0.008, HR 3.570, 95%CI 1.384–9.209). Our data demonstrated that *DNMT3A* mutations were important OS and EFS factors for *FLT3-*ITD mutated AML patients (Figs 1A and 1B). *DNMT3A* mutations positivity adversely affect transplantation outcomes after HSCT in *FLT3-*ITD mutated AML patients. *DNMT3A* mutations adversely affected both OS and EFS in these patients (Fig. 1A and 1B), and landmark analysis showed that *DNMT3A* mutated cases had markedly shorter median OS (28 vs. 44 months), EFS (20 vs. 44 months), and RFS (16 vs. 41 months) compared with *DNMT3A*–wild-type patients (P = 0.001 for all). For *RUNX1* mutations, *FLT3-ITD* mutated AML patients had lower EFS rate than without *RUNX1* mutations, as *FLT3-ITD* mutated AML patients tend to have lower OS rate than those without *RUNX1* mutations. We speculated that *DNMT3A* mutations and *RUNX1* mutations pay essential roles in these patients’ relapse and drug resistance.

### 3.4 *HLF* high expression predicts poor OS and drug resistance

**Fig 2:**
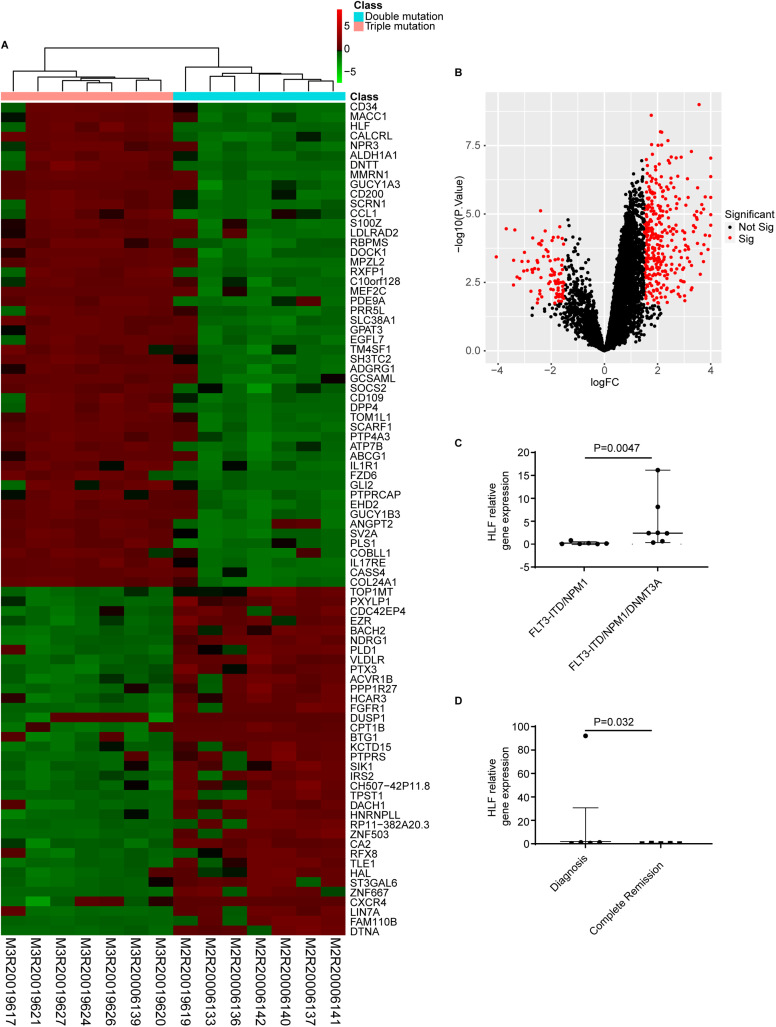
Gene expression in FLT3-ITD mutated AML patients. A: Genes showing over- or under-expression in the heatmap are shown in red or green, respectively. B: Volcano plots showing DEGs in triple-mutated and double-mutated AML patients. C: HLF gene expression in triple-mutated and double-mutated AML patients. D: HLF gene expression in triple-mutated AML patients at the time of diagnosis and complete remission after patients treated with new FLT3-ITD inhibitor sorafenib.

RNA sequencing of newly diagnosis samples were performed in 7 patients who had *FLT3-*ITD*/NPM1* without *DNMT3A* mutations as well as 7 patients who had *FLT3-*ITD*/NPM1* coexist with *DNMT3A* mutations. The gene expression profile of bone marrow mononuclear cells in triple-mutated AML patients and that of double-mutated AML had statistical difference in 1633 gene. Among them, the expression of *HLF* gene increased in triple-mutated AML patients (Figs 2A and 2B).

To further identify the role of *HLF* gene in *FLT3-*ITD mutated AML patients, we tested *HLF* gene expression in triple and double-mutated AML patient. We found the *HLF* gene expression was higher in triple-mutated AML patients than that of double-mutated AML patients (P = 0.0047, Fig 2C). We further validated this finding using the TCGA database (version 2024-08-05); however, due to the limited number of cases, the difference in *HLF* expression between triple- and double-mutated patients did not reach statistical significance (P = 0.401, S5 Table, S1 Fig). We also verified the *HLF* gene expression in triple-mutated AML patients at the time of newly diagnosis and CR after patients treated with new *FLT3*-ITD inhibitor sorafenib. We analysis that the expression of *HLF* gene was lower in triple-mutated AML patients treated with new *FLT3-*ITD inhibitor sorafenib than that of double-mutated AML patients at newly diagnosis (P = 0.032, Fig 2D). Furthermore, we inferred that overexpression of *HLF* gene may negatively impact the relapse and drug resistance of *FLT3-*ITD mutated AML patients.

## 4. Discussion

In this study, we investigated the clinical and molecular characteristics of 84 *FLT3‐*ITD mutated AML patients and identified that high *HLF* expression was strongly associated with poor overall survival and increased drug resistance, particularly in the subgroup harboring concurrent *DNMT3A* mutations. Our data demonstrated that *DNMT3A* mutations significantly worsened prognosis in *FLT3‐*ITD mutated AML, reducing both CR rates after standard chemotherapy and long‐term survival outcomes. RNA sequencing further revealed that *HLF* overexpression was enriched in triple‐mutated cases, suggesting a potential mechanistic link between *DNMT3A* mutations, *HLF* dysregulation, and chemoresistance. These findings highlight *HLF* as a novel prognostic biomarker and potential therapeutic target in this high‐risk AML subtype, which requires confirmation in future studies.

*FLT3*-ITD mutation was recognized as one of driver gene mutations in adult AML patients [19]. The biological mechanisms which *FLT3*-ITD mutations contribute to leukemogenesis are still not clear. In the past decade, *FLT3*-ITD mutations have been attracting much attention as a marker for risk stratification and a poor prognostic factor in adult AML patients [20]. High-dose chemotherapy and HSCT could improve prognosis of most AML patients, but not include *FLT3* mutated AML patients, clinical development of *FLT3* kinase inhibitors expected. However, with the application of *FLT3* inhibitors, resistance of *FLT3* inhibitors have already become apparent [21]. The resistance mechanisms are complex and emerging resistant clones are heterogenous [22]. Further basic and clinical studies are required to establish the best therapeutic strategy for AML patients with *FLT3* mutations [23].

In our cohort, no significant difference was found in the OS, EFS and relapse incidence between *FLT3*-ITD mutated AML patients with and without NPM1 mutations, while significant difference was found in the OS, EFS and relapse incidence between *FLT3*-ITD mutated AML patients with and without *DNMT3A* mutations. Consistent with our previous research results of clinical medical center that *DNMT3A* R882 mutation was an unfavorable prognostic marker in *FLT3*-ITD mutated AML patients treated with HSCT [24,25]. Contrary to our results, several studies have shown that HSCT cannot abrogate the unfavorable effect of *FLT3*-ITD mutations in AML patients [26]. In *FLT3*-ITD mutated AML patients, *DNMT3A* mutated patients had lower CR rate after one and two standard chemotherapy than that of patients without *DNMT3A* mutations (P = 0.073, 0.028, respectively).

Prior studies support these observations. Guryanova et al. reported that *DNMT3A* mutations induced hematopoietic stem cell expansion, cooperated with mutations in the FMS-like tyrosine kinase 3 gene and the nucleophosmin gene to induce AML in vivo, and promoted resistance to anthracycline chemotherapy [14]. These findings highlight a possible association between DNMT3A mutations and chemoresistance, meriting further study. Jonathan bond reported that *DNMT3A* mutations strongly correlated with disease relapse and short survival, and these prognostic effects were independent of patients’ age [27]. Our RNA‐seq data add that triple‐mutated AML cases exhibit distinct gene expression profiles, including significant *HLF* overexpression. These identifies a correlation between *HLF* overexpression and resistance, we recognize that causal mechanisms have not yet been established. Currently, no in vitro or mechanistic experiments have been conducted to directly support this link.

The biological role of *HLF* in AML, particularly in poor‐prognosis subtypes such as *FLT3‐*ITD–mutated AML, remains largely unexplored. Given its association with both *DNMT3A* mutation status and adverse outcomes in our study, *HLF* may contribute to chemoresistance and disease progression. Targeting *HLF*‐driven transcriptional programs could therefore represent a novel therapeutic strategy for this challenging AML subset.

## 5. Conclusion

To our knowledge, the clinical significance of *DNMT3A* mutation in triple‐mutated AML remains unclear, although it is associated with poor prognosis in *FLT3-ITD*‐mutated AML patients undergoing HSCT. Future studies will explore the association between *HLF* expression and drug resistance in triple‐mutated AML.

## Supporting information

S1 Raw Figures Raw data for S1 figure. (XLS)

S1 Fig *HLF* gene expression in triple-mutated and double-mutated AML patients in TCGA database. (TIF)

S1 Table The matching detection includes 51 common hot spot genes related to hematological tumors. (DOCX)

S2 Table Validation of FLT3-ITD improved examination in cell line. (DOCX)

S3 Table Validation of FLT3-ITD improved examination in bone marrow samples. (DOCX)

S4 Table Clinical characteristics of patients with FLT3-ITD mutations. (XLSX)

S5 Table Validation of *HLF* gene expression in TCGA database. (XLSX)
