# Supplementary figures and images for: HLF gene is a poor prognostic factor in acute myeloid leukemia patients with FLT3-ITD/NPM1 mutations undergoing hematopoietic transplantation
Source: PLoS One. 2025 Oct 14;20(10):e0333690. doi: 10.1371/journal.pone.0333690 (PMC12520370; doi:10.1371/journal.pone.0333690)

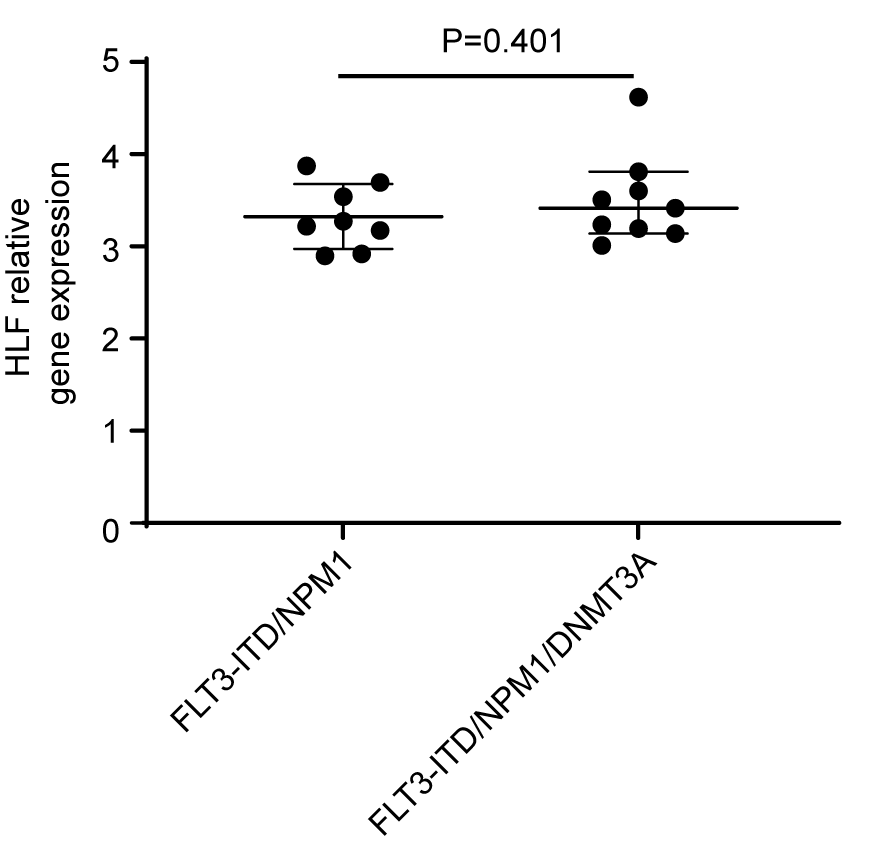

Supplement: S1 Fig — (TIF) [file pone.0333690.s002.tif]
