# Supplementary material for: HLF gene is a poor prognostic factor in acute myeloid leukemia patients with FLT3-ITD/NPM1 mutations undergoing hematopoietic transplantation
Source: PLoS One. 2025 Oct 14;20(10):e0333690. doi: 10.1371/journal.pone.0333690 (PMC12520370; doi:10.1371/journal.pone.0333690)
Supplement: S1 Table — (DOCX) [file pone.0333690.s003.docx]

**Supplementary table 1** the matching detection includes 51 common hot spot genes related to hematological tumors

| *ASXL1* | *CEBPA* | *FLT3* | *MPL* | *PIGA* | *STAG2* |
| --- | --- | --- | --- | --- | --- |
| *ASXL2* | *c-kit* | *GATA2* | *MYD88(L265P)* | *PTEN* | *TET2* |
| *BCOR* | *CSF3R* | *IDH1* | *NOTCH1* | *PTPN11* | *TP53* |
| *BCORL1* | *CSMD1* | *IDH2* | *NPM1* | *RUNX1* | *U2AF1* |
| *BIRC3* | *DNMT3A* | *IL7R* | *NRAS* | *SETBP1* | *WT1* |
| *BRAF* | *ETNK1* | *JAK1* | *PAX5* | *SETD2* | *ZRSR2* |
| *CALR* | *ETV6* | *JAK2* | *PDGFRA* | *SF3B1* |  |
| *CBL* | *EZH2* | *JAK3* | *PDGFRB* | *SH2B3* |  |
| *CDKN2A(P16)* | *FBXW7* | *KRAS* | *PHF6* | *SRSF2* |  |
