# Supplementary material for: HLF gene is a poor prognostic factor in acute myeloid leukemia patients with FLT3-ITD/NPM1 mutations undergoing hematopoietic transplantation
Source: PLoS One. 2025 Oct 14;20(10):e0333690. doi: 10.1371/journal.pone.0333690 (PMC12520370; doi:10.1371/journal.pone.0333690)
Supplement: S2 Table — (DOCX) [file pone.0333690.s004.docx]

**Supplementary table 2** Validation of *FLT3*-ITD improved examination in cell line

| MV4-11: K562 AR | Sample number | conventional method | improved method |
| --- | --- | --- | --- |
| 0.1 | 4 | 4 | 4 |
| 0.01 | 8 | 4 | 8 |
| 0.001 | 8 | 2 | 8 |
| 0.0005 | 8 | 0 | 2 |
| 0.0001 | 8 | 0 | 0 |
