# Supplementary material for: HLF gene is a poor prognostic factor in acute myeloid leukemia patients with FLT3-ITD/NPM1 mutations undergoing hematopoietic transplantation
Source: PLoS One. 2025 Oct 14;20(10):e0333690. doi: 10.1371/journal.pone.0333690 (PMC12520370; doi:10.1371/journal.pone.0333690)
Supplement: S3 Table — (DOCX) [file pone.0333690.s005.docx]

**Supplementary table 3** Validation of *FLT3*-ITD improved examination in bone marrow samples

| ITD | Sample AR | Sample number | conventional method | improved method |
| --- | --- | --- | --- | --- |
| 57bp | 0.01 | 8 | 8 | 8 |
|  | 0.001 | 8 | 0 | 8 |
|  | 0.0005 | 8 | 0 | 2 |
|  | 0.0001 | 8 | 0 | 0 |
| 108bp | 0.01 | 8 | 8 | 8 |
|  | 0.001 | 8 | 0 | 8 |
|  | 0.0005 | 8 | 0 | 1 |
|  | 0.0001 | 8 | 0 | 0 |
